# Clinical Characterization of Parkinson's Disease Patients With Cognitive Impairment

**DOI:** 10.3389/fneur.2020.00731

**Published:** 2020-08-04

**Authors:** Ana Simon-Gozalbo, Carmen Rodriguez-Blazquez, Maria J. Forjaz, Pablo Martinez-Martin

**Affiliations:** ^1^Doctorate Program in Health Sciences, University of Alcala, Alcala de Henares, Spain; ^2^National Center of Epidemiology and CIBERNED, Carlos III Institute of Health, Madrid, Spain

**Keywords:** cognitive dysfunction, Parkinson's disease, motor and non-motor symptoms, dementia, mild cognitive impairment, clinical characteristics

## Abstract

**Background:** Cognitive impairment is one of the most frequent and disabling non-motor symptoms in Parkinson disease (PD) and encompasses a continuum from mild cognitive impairment (PD-MCI) to dementia (PDD). The risk factors associated with them are not completely elucidated.

**Objective:** To characterize the presence and clinical presentation of PD-MCI and PDD in patients with idiopathic PD, examining motor and non-motor features and determining factors associated with cognitive impairment.

**Methods:** Multicenter, cross-sectional study in 298 PD patients who underwent clinical [Hoehn and Yahr (HY) staging and Clinical Impression of Severity Index for Parkinson Disease], neurological [Scales for Outcomes in Parkinson's Disease (SCOPA)-Motor], neuropsychological (Mini Mental State Examination, SCOPA-Cognition, Frontal Assessment Battery and Clinical Dementia Rating Scale), neuropsychiatric [SCOPA-Psychiatric complications, SCOPA-Psychosocial (SCOPA-PS), and Hospital Anxiety and Depression Scale (HADS)], and health-related quality of life [Parkinson Disease Questionnaire for quality of life (PDQ-8)] assessment. Movement Disorders Society criteria were applied to classify patients as normal cognition (NC), PD-MCI, and PDD. The association between variables was explored using multivariate binary and multinomial logistic regression models.

**Results:** Seventy-two patients (24.2%) were classified as NC, 82 (27.5%) as PD-MCI, and 144 (48.3%) as PDD. These last two groups reported more psychosocial problems related with the disease (mean SCOPA-PS, 16.27 and 10.39, respectively), compared with NC (7.28) and lower quality-of-life outcomes (PDQ-8 48.98 and 28.42, respectively) compared to NC (19.05). The logistic regression analysis showed that both cognitive impaired groups had a more severe stage of PD measured by HY [odds ratio (OR) for MCI-PD, 2.45; 95% confidence interval (CI), 1.22–4.90; OR for PDD 2.64; 95% CI, 1.17–5.98]. Specifically, age (OR, 1.30; 95% CI, 1.16–1.47), years of education (OR, 0.91; 95% CI, 0.83–0.99), disease duration (OR, 1.19; 95% CI, 1.07–1.32), HADS-D (OR, 1.20; 95% CI, 1.06–1.35), and hallucinations (OR, 2.98; 95% CI, 1.16–7.69) were related to PDD.

**Conclusions:** Cognitive impairment in PD is associated with more severe disease stage, resulting in a global, neuropsychiatric, psychosocial, and quality-of-life deterioration. This study provides a better understanding of the great impact that cognitive impairment has within the natural history of PD and its relationship with the rest of motor and non-motor symptoms in the disease.

## Background

Idiopathic Parkinson disease (PD) is a common, chronic, and neurodegenerative disorder characterized by the presence of motor manifestations such as tremor, rigidity, bradykinesia, or instability. However, there are also non-motor features including cognitive dysfunction, sleep disorders, neuropsychiatric symptoms, autonomic dysfunction, pain, fatigue, and olfactory disorders that may be present since the earliest stages of PD or even prior to its diagnosis, generating a great impact on patients and caregivers (1, 2). In conjunction with this fact, the latest evidence from clinical, genetic, neuropathological, and imaging studies suggests the initiation of PD-specific pathology prior to the initial presentation of the classical motor clinical features by years (preclinical and prodromal stages of PD). The existence of these “premotor biomarkers” opens up the possibility to new therapies that would help prevent the onset of the disease or retard the progression (3). One of the most recent neuroprotective substances would be the cystatin C, which seems to play a significant role in neural and vascular cell function in neurodegenerative diseases (4, 5). Another new biomarker related to PD progression could be the decreased serum levels of mitochondrial creatine kinase. However, no significant relationship was found between this levels and the Hoehn and Yahr (HY) stage or non-motor symptoms scales (6).

With regard to cognition, the heterogeneous presentations encompass a continuum from cognitively intact patients to subjective cognitive decline, mild cognitive impairment (PD-MCI), and, finally, PD dementia (PDD), with a progressive severity gradient (7). Cognitive decline is one of the most frequent clinical manifestations of PD, being a prognostic variable of institutionalization and mortality (8). Prevalence of PD-MCI is estimated between 15 and 40% of PD patients according to recent studies and ~40% of patients with PD-MCI decline to PDD over 3 years (9). In 2007 and 2012, the Movement Disorder Society (MDS) published the diagnostic criteria PDD and PD-MCI, respectively. For both disorders, MDS established two levels of diagnostic certainty: level I, using a brief neuropsychological evaluation; and level II, which includes a much more extensive battery of tests (10, 11).

Several sociodemographic and disease-related features have been identified as potential risk factors that increase the progression of cognitive decline in PD, such as old age, low educational level, severity of disease, high age at onset, disease duration, high doses of levodopa, or use of anticholinergic medication (12, 13). Other clinical manifestations, such as neuropsychiatric symptoms, hallucinations, or rapid eye movement–sleep behavior disorder (RBD), have also been investigated and even considered as prodromal markers of PD (3, 14). The pathophysiological mechanisms underlying RBD and its relation with PDD include a major cholinergic denervation, a higher burden of cerebrovascular disease, and a more advanced deposition of synaptic nucleoprotein in brain areas (15). With regard to the link between depression symptoms and different types of dementia, some animal models have been developed, showing that exposure to several exogenous factors (economic status, education, family support, and social environment) and endogenous factors (such as aging, cerebral small vessel disease, brain circuits, neuroendocrine activity, neurochemistry, neurotransmitters, and inflammatory cytokines) could contribute to the pathogenesis of depression. Overall, non-motor symptoms in PD patients have been associated with dysfunction of the microbiota–gut–brain axis (16). Specifically, PDD has been associated to certain biomarkers such as low cerebrospinal fluid levels of β-amyloid 42, low serum uric acid levels, low serum Trefoil factor 3 levels, low serum cholinesterase activity, and high serum levels of homocysteine (17). In addition, some proinflammatory substances such as lipoprotein-associated phospholipase A_2_, superoxide dismutase, and high-sensitivity C-reactive protein are linked to neuroinflammation and therefore to progression of disease and cognitive impairment (18–20).

Concerning PD-MCI status, there are only a few studies on associated risk factors, and they suggest that older age, late age at onset, male gender, depression, and more advanced motor impairment have been associated with PD-MCI (9, 21). However, when reviewing the existing literature, the results are still conflicting regarding some of these factors. For example, contradictory findings have been reported for gender, education, age at onset, disease duration, HY stage, depression, and levodopa dose (12, 13, 22, 23). It is suggested that the reason for this is due to differences in populations, differences in methodology, outcome measures, and lack of robust studies with large sample size (24). In neuroimaging studies, PD-MCI patients showed more severe atrophy in the right entorhinal cortex, compared to PD patients without cognitive impairment, suggesting this brain area as a neuroanatomical biomarker in PD-MCI (25).

In the last decades, studies have tried to achieve a better understanding of the clinical heterogeneity in PD by defining different subtypes within the disease via cluster analysis, which could predict disease course, underlie neuropathological mechanisms, and lead to more efficient, personalized, therapeutic strategies (26, 27). The traditional subtyping systems were based on motor symptoms and motor complications, such as tremor-dominant PD (TD) vs. postural instability and gait difficulty (PIGD) with an akinetic-rigid predominance (28, 29). The TD motor phenotype was considered to have a more favorable prognosis than the PIGD phenotype, whereas the latter is associated to a more rapid and greater progression of the disease, including cognitive impairment (30, 31). Several studies have defined subtypes in relation to demographic and disease-related factors, such as the age at onset, claiming that patients with young-onset PD had a slower progression of disease than those with late-onset PD, concluding that age at onset was a major determinant of the course of disease (32). In recent years, the increasing importance of non-motor symptoms in PD has led to non-motor symptoms based subtypes, such as cognitive PD phenotypes (33, 34). However, the clinical, neuropsychological, and neuropathological boundaries between PDD and dementia with Lewy bodies (DLBs) have challenged the concept of different clinical entities. Conventionally, the diagnosis of PDD is usually made when dementia develops within the context of an established PD, whereas DLBs might be more appropriate when dementia precedes or coincides within 1 year of Parkinsonism onset (35). The so-called “Park cognitive” subtype is characterized by developing cognitive impairment even at an early stage, progressing rapidly to dementia (36). In other cognitive models graded by severity of cognitive impairment, cognitively intact patients were significantly younger and had received more years of formal education, whereas patients in the more cognitive impaired clusters had more severe motor symptoms, longer disease duration, and more axial signs (7).

Finally, the latest cluster analysis includes clinical, neuropsychological, neuroimaging, biospecimen, and genetic information to develop criteria to assign patients to a PD subtype (30, 37). The so-called “diffuse malignant” phenotype, in which three critical non-motor features (MCI, RBD, and orthostatic hypotension) at baseline identified the most rapidly progressive subtype, showed more severe motor and non-motor symptoms, more atrophy in substantia nigra–connected areas, more dopaminergic deficit on SPECT, reduced β-amyloid in cerebrospinal fluid, and shorter survival rates (38). Moreover, this subtype had greater decline in cognition and in dopamine functional neuroimaging after an average of 2.7 years (30).

However, subtyping PD has been challenging, because of inconsistent reliability and possibility of confusion between subtypes and different stages of disease progression (27). Therefore, there is currently no clear way to define and divide subtypes in PD.

The aim of the present study is to examine and compare the sociodemographic, disease-related, and clinical characteristics in a sample of PD patients with different degrees of cognitive impairment determined using the MDS diagnostic criteria. Additionally, a detailed analysis of the relationship between cognitive decline in PD and other manifestations of the disease will be carried out. The aforementioned aspects are not fully understood and are key to the management and well-being promotion of these patients. Moreover, these clinical markers have still potential to be used alongside with other biological and neuroimaging biomarkers as indicators of cognitive impairment (39).

## Methods

This study used data from a previous international, multicenter, cross-sectional study (40). Patients 30 years or older and diagnosed with idiopathic PD according to the UK Parkinson's Disease Society Brain Bank Criteria were included (41). To obtain an adequate sample size of at least 100 patients with PDD, patients with cognitive impairment were specifically overrecruited. Exclusion criteria were as follows: (1) parkinsonism other than idiopathic PD; (2) acute or chronic concomitant disease that could interfere in the evaluation of PD; and (3) any type of disability that could interfere with answering the questionnaires, with the exception of cognitive impairment. In cognitive impaired patients, information pertaining to self-administered and patient-reported outcome (PRO) scales was obtained from clinical interview to the caregiver.

Patients were recruited from movement disorder clinics in Brazil (*n* = 76), Ecuador (*n* = 95), and Romania (*n* = 127). A neurologist with expertise in movement disorders collected sociodemographic and disease-related data. In addition, a neurological, neuropsychological, neuropsychiatric, functional, and quality-of-life assessment was carried out, applying different rating scales: the global severity of PD was defined according to the HY staging (42) and the Clinical Impression of Severity Index (CISI-PD) (43); functional status was assessed through the Barthel Index (44); the motor manifestations were evaluated by the Scales for Outcomes in Parkinson's Disease-Motor (SCOPA-Motor) (45); the assessment of cognitive status included the Mini-Mental State Examination (MMSE) (46), the SCOPA-Cognition (SCOPA-Cog) (47), the Frontal Assessment Battery (FAB) (48, 49), and the Clinical Dementia Rating Scale (CDR) (50); the neuropsychiatric symptoms were collected through the SCOPA-Psychiatric (SCOPA-PC) (51). Finally, each patient completed the following self-administered questionnaires: the Hospital Anxiety and Depression Scale (HADS), with two subscales, HADS-A and HADS-D, respectively (52); the 8-item Parkinson Disease Questionnaire for quality of life (PDQ-8) (53); and the SCOPA-Psychosocial (SCOPA-PS) for psychosocial consequences of PD (54). For all instruments except cognitive tests (MMSE, SCOPA-Cog, and FAB) and the Barthel Index, higher scores reflect poorer functioning. A cutoff of ≥11 in the HADS-A and HADS-D was used as indicative of presence of anxiety and depression (52). All evaluations were performed during “on” state. The culturally adapted and validated versions were used in each country.

Neurologists evaluated the severity of cognitive impairment using the neuropsychological examination and the clinical interview with patient and caregiver, CDR scale scores, the presence or absence of dementia according to *Diagnostic and Statistical Manual of Mental Disorders, Fourth Edition* criteria, and item 3 of the CISI-PD scale, which refers to the patient's cognitive status. Cognitive symptoms started at least 1 year after the onset of PD in all participants.

Subsequently and independently, patients were classified by the research team into three groups, namely, normal cognition (NC), PD-MCI, and PDD, using criteria based on those proposed by the MDS (level I) and literature review [Table 1; (10, 11, 40, 55)].

**Table 1:** Classification criteria based on MDS criteria and literature review.

	**Main criteria**	Secondary criteria (for doubtful cases)*
PDD	1 Clinical criteria: - CDR ≥1 - DSM IV compatible with dementia - CISI-PD cognitive status ≥3 + Objective criteria: - MMSE <26 + ≥2 cognitive domains affected	≥2 Clinical criteria: - CDR ≥1 - DSM IV compatible with dementia - CISI-PD cognitive status ≥3
PD-MCI	1 Clinical criteria: - CDR = 0.5 - CISI-PD cognitive status = 1–2 + Objective criteria: - SCOPA Cog 17–23	2 Clinical criteria: - CDR = 0.5 - CISI-PD cognitive status = 1–2
NC	1 Clinical criteria: - CDR = 0 - CISI-PD cognitive status = 0 + Objective criteria: - SCOPA Cog ≥24	≥2 Clinical criteria: - CDR=0 - CISI-PD cognitive status = 0

PDD, Parkinson's disease dementia; PD-MCI, Parkinson's disease-Mild cognitive impairment; NC, normal cognition; CDR, Clinical Dementia Rating; DSM-IV, Diagnostic and Statistical Manual of Mental Disorders, fourth edition; CISI-PD, Clinical Impression of Severity Index-Parkinson's disease; MMSE, Mini Mental State Examination; SCOPA-Cog, Scales for Outcomes in Parkinson's disease-Cognition. *Cases that did not fulfill all the criteria.

The study was formally approved by the local institutional review boards. Written informed consent was obtained from all patients prior to participating in the study. In those with severe dementia, informed consent was signed by the main caregiver.

### Statistical Analysis

In addition to descriptive statistics, tests for determining the differences between the 3 groups of patients were used. For data not fitting the normal distribution, non-parametric tests were applied. Chi-square χ^2^-tests were used for comparing categorical variables, whereas the Kruskal–Wallis test was applied in continuous variables that were not normally distributed.

Two multivariate binary logistic regression models were subsequently performed: one with the presence of any degree of cognitive impairment (NC vs. MCI-PD/PDD) and the other one with the presence of dementia (NC/MCI-PD vs. PDD), as dependent variables. The independent variables were motor and non-motor symptoms variables and time since diagnosis in years. Moreover, a multinomial logistic regression model was undertaken, with the same independent variables and using the three diagnostic groups as the outcome variable. Variables with *p* < 0.10 from the univariate analysis and those that were considered clinically relevant were selected for inclusion in the multivariate analyses. Age, sex, and education were controlled. Odds ratio (OR) with 95% confidence intervals (CIs) was used to assess the significance of associations. The binary logistic regression models were carried out to help clarifying the interpretation of data in the two stages of cognitive decline (MCI-PD and PDD) and, at the same time, adding more information to the topic, contrasting and completing the data provided by the multinomial logistic regression model.

In the logistic regression models, the cognitive assessment tests were not included because they had been used for the classification of the diagnostic groups and therefore could interfere in the results of the analysis. To analyze the relationship between the different variables, Spearman correlation coefficients were determined. Coefficients of ≥0.60 were considered as collinearity. According to this test, the CISI-PD was not included, because of collinearity with the SCOPA-Motor scale and the HY. Likewise, the SCOPA-PC was not introduced in the models, as it may have interactions with hallucinations (an item from the scale) and HADS. Finally, because the age at onset is dependent on the patient's baseline age and the disease duration, it was also excluded.

Statistical calculations were performed with the Statistical Package for the Social Sciences version 22.0 IBM (Armonk, New York).

## Results

### Sociodemographic and Disease-Related Characteristics

The sample was composed of 298 patients (186 men and 112 women). Applying the MDS criteria, 72 participants (24.2%) were classified as NC, 82 (27.5%) as PD-MCI, and 144 (48.3%) as PDD (Table 2).

**Table 2:** Sociodemographic and disease-related features of study groups.

	**Total (298)**	**95% CI**	**NC (72)**	**PD-MCI (82)**	**PDD (144)**	p*
Age, year	68.06 (9.62)	66.96–69.16	63.26 (10.17)	63.90 (9.46)	72.83 (6.78)	<0.001^a^
Sex, % men	62.40		65.30	52.40	66.70	0.089^b^
Education, year	8.70 (4.41)	8.20–9.21	11.19 (4.08)	9.23 (4.35)	7.16 (3.98)	<0.001^a^
Age at onset, year	58.49 (10.12)	57.34–59.65	56.99 (10.98)	56.35 (11.77)	60.47 (8.17)	0.026^a^
Disease duration, year	9.57 (6.47)	8.83–10.31	6.28 (4.26)	7.55 (4.62)	12.36 (7.09)	<0.001^a^
Treatment, %						
L-dopa	62.80		59.70	64.60	63.20	0.811^b^
Agonists	18.10		33.30	25.60	6.30	<0.001^b^
Marital status, % married	68.80		73.60	72.00	64.60	0.309^b^
Employment, % employees	17.10		47.20	15.90	2.80	<0.001^b^

Continuous variables are expressed as means (standard deviations). Categorical variables are expressed as percentages. *Differences are calculated with

a Kruskal-Wallis test and

b *Chi-square test. CI, confidence interval; NC, normal cognition; PD-MCI, Parkinson's disease-Mild cognitive impairment; PDD, Parkinson's disease dementia*.

The mean age of the total sample was 68.06 [standard deviation (*SD*) = 9.62] years with 8.7 (*SD* = 4.41) years of education. Parkinson disease MCI and PDD patients were significantly older and presented a lower education level than NC patients. No differences by sex were found between the three cognition groups. Overall, age at onset was 58.49 (*SD* = 10.12) years, with a statistically significantly higher age at onset in the PDD group than the other groups. The average duration of the disease at the time of data collection was 9.57 (*SD* = 6.47) years, being significantly higher in the cognitive impaired groups (12.36 for PDD vs. 6.28 years for NC). Dopamine agonists were more frequently taken in NC patients (33.3% in the intact vs. 6.3% in the PDD group, *p* < 0.05), and there were no significant differences regarding l-dopa treatment between the groups.

### Motor and Non-motor Symptoms

Parkinson disease MCI and PDD patients presented a more severe stage of disease (according to HY), as well as a worse clinical, motor, and functional status (measured by the CISI-PD, SCOPA-Motor, and Barthel index, respectively) than NC patients. Regarding the cognitive sphere, as expected, all cognitive test scores were worse in the cognitively impaired groups, with the lowest scores for PDD patients. The latter also obtained the highest average score on the neuropsychiatric symptom scale (SCOPA-PC). Specifically, 43.8% of PDD patients exceeded the cutoff point for anxiety, and 55.6% surpassed it for depression. One-third of PDD patients (34%) had hallucinations, compared to only 8 and 5% in PD-MCI and NC, respectively. There were lower quality-of-life and more psychosocial consequences in the cognitively impaired groups. All tests showed significant differences (*p* < 0.05) between the three study groups (Table 3).

**Table 3:** Motor and non-motor symptoms in study groups.

	**NC (*n* = 72)**	**PD-MCI (*n* = 82)**	**PDD (*n* = 144)**	***p***
Hoehn and Yahr stage	2 (1–2)	3 (2–3)	3 (3–4)	<0.001
CISI-PD	7.00 (4.29)	10.06 (3.62)	16.36 (3.39)	<0.001
Barthel Index	97.22 (10.51)	87.65 (17.74)	63.23 (28.44)	<0.001
SCOPA-Motor, total score	15.96 (10.05)	22.29 (10.96)	36.42 (13.50)	<0.001
Motor examination	8.57 (5.26)	12.13 (6.09)	19.35 (7.93)	<0.001
Activities of daily living	5.31 (3.28)	6.91 (3.51)	11.73 (4.40)	<0.001
Motor complications	2.08 (3.13)	3.24 (2.90)	5.34 (3.42)	<0.001
MMSE, total score	29.06 (1.26)	27.22 (2.31)	16.24 (6.85)	<0.001
SCOPA-Cog, total score	30.14 (5.87)	22.80 (5.17)	9.69 (5.67)	<0.001
Memory	12.86 (4.29)	8.68 (3.41)	3.88 (2.60)	<0.001
Attention	3.83 (0.47)	3.41 (1.03)	1.23 (1.45)	<0.001
Executive functions	9.42 (1.79)	7.48 (1.93)	3.27 (2.18)	<0.001
Visuospatial functions	4.03 (0.85)	3.23 (1.03)	1.32 (1.36)	<0.001
FAB, total score	15.56 (2.59)	13.09 (2.83)	6.63 (3.99)	<0.001
SCOPA-PC, total score	4.11 (3.36)	3.27 (3.63)	5.84 (5.25)	<0.001
Hallucinations, %	5.60	8.50	34.00	<0.001
HADS, Anxiety	6.42 (4.19)	8.51 (3.85)	9.77 (4.46)	<0.001
Patients with anxiety, %	20.80	29.30	43.80	0.002
HADS, Depression	6.08 (4.18)	7.63 (3.82)	10.36 (4.22)	<0.001
Patients with depression, %	16.70	15.90	55.60	<0.001
PDQ-8	19.05 (16.74)	28.42 (22.44)	48.98 (22.22)	<0.001
SCOPA-PS, total score	7.28 (5.45)	10.39 (6.40)	16.27 (7.03)	<0.001

*Continuous variables are expressed as means (standard deviations). Hoehn and Yahr stage is expressed as median (interquartile range). Categorical variables are expressed as percentages. Differences were calculated with Kruskal-Wallis test in continuous variables and Chi-square test in categorical variables. NC, normal cognition; PD-MCI, Parkinson's disease-Mild cognitive impairment; PDD, Parkinson's disease dementia; CISI-PD, Clinical Impression of severity Index for Parkinson's Disease; SCOPA-Motor, Scales for Outcomes in Parkinson's Disease-Motor; MMSE, Mini Mental State Examination; SCOPA-Cog, SCOPA-Cognition; FAB, Frontal Assessment Battery; SCOPA-PC, SCOPA-Psychiatric Complications; HADS, Hospital Anxiety and Depression Scale; PDQ-8, 8-item Parkinson's Disease Questionnaire; SCOPA-PS, SCOPA-Psychosocial*.

### Association Between Cognitive Impairment and Motor and Non-motor Symptoms

In the first regression model (Table 4), with cognitive impairment (PD-MCI or PDD) as dependent variable, two variables were found to be significant. Hoehn and Yahr stage (OR, 2.06; 95% CI, 1.10–3.85) and the motor exploration subscale in SCOPA-Motor scale (OR, 1.11; 95% CI, 1.01–1.21) showed a positive association with cognitive dysfunction. This model explained 49.0% of the variance.

**Table 4:** Binary logistic regression model for normal cognition vs. cognitive impairment (PD-MCI/PDD).

	**Univariate analysis**			**Multivariate analysis**		
	**Crude OR**	**95% CI**	***p***	**Adjusted OR**	**95% CI**	***p***
Sex (women)	1.17	0.67–2.04	0.56	1.51	0.74–3.09	0.255
Age	1.07	1.04–1.10	** <0.001**	1.01	0.97–1.05	0.411
Age at onset	1.02	0.99–1.05	0.148	–	–	–
Years of education	0.84	0.79–0.90	** <0.001**	0.9	0.83–0.98	**0.013**
Disease duration	1.18	1.10–1.27	** <0.001**	1.07	0.96–1.18	0.204
Hoehn and Yahr	4.28	2.94–6.23	** <0.001**	2.06	1.10–3.85	**0.022**
Barthel Index	0.9	0.87–0.94	** <0.001**	0.94	0.90–0.97	**0.002**
SMS-Motor examination	1.19	1.13–1.25	** <0.001**	1.11	1.01–1.21	**0.019**
SMS-ADL	1.31	1.21–1.42	** <0.001**	0.86	0.72–1.03	0.101
SMS-Motor complications	1.29	1.16–1.42	** <0.001**	0.87	0.72–1.04	0.137
SCOPA-PC	1.04	0.98–1.10	0.199	–	–	–
Hallucinations^*^	5.6	1.95–16.04	0.001	1.57	0.41–5.99	0.504
HADS, Anxiety	1.17	1.09–1.25	** <0.001**	1,00	0.89–1.13	0.904
HADS, Depression	1.21	1.12–1.30	** <0.001**	1.07	0.95–1.21	0.213

*OR, odds ratio; CI, confidence interval; SMS, Scales for Outcomes in Parkinson's disease-Motor Scale; ADL, activities of daily living; SCOPA-PC, Scales for Outcomes in Parkinson's disease-Psychiatric Complications; HADS, Hospital Anxiety and Depression Scale*.

* *Hallucinations: item from SCOPA-PC. Bold indicates the significant values*.

The second regression model (Table 5), with dementia as dependent variable, showed that dementia was positively associated with higher age (OR, 1.30; 95% CI, 1.16–1.47) and disease duration (OR, 1.19; 95% CI, 1.07–1.32), an increased score in HADS-D (OR, 1.20; 95% CI, 1.06–1.35), and more hallucination symptoms (OR, 2.98; 95% CI, 1.16–7.69). The motor complications SCOPA-Motor subscale (OR, 0.82; 95% CI, 0.69–0.98) was negatively associated with the presence of dementia. This model explained 63.5% of the variance.

**Table 5:** Binary logistic regression model for dementia vs. no dementia (NC/PD-MCI).

	**Univariate analysis**			**Multivariate analysis**		
	**Crude OR**	**95% CI**	***p***	**Adjusted OR**	**95% CI**	***p***
Sex (women)	0.70	0.44–1.12	0.144	0.76	0.37–1.54	0.455
Age	1.15	1.11–1.20	** <0.001**	1.30	1.16–1.47	** <0.001**
Age at onset	1.04	1.01–1.07	** <0.001**	–	–	–
Years of education	0.84	0.79–0.89	** <0.001**	0.91	0.83–0.99	**0.044**
Disease duration	1.18	1.12–1.25	** <0.001**	1.19	1.07–1.32	**0.001**
Hoehn and Yahr	5.56	3.67–8.43	** <0.001**	1.45	0.75–2.80	0.26
Barthel Index	0.94	0.92–0.95	** <0.001**	0.97	0.95–0.99	**0.038**
SMS-Motor examination	1.18	1.14–1.23	** <0.001**	1.05	0.98–1.14	0.134
SMS-ADL	1.41	1.30–1.53	** <0.001**	0.99	0.84–1.18	0.967
SMS-Motor complications	1.27	1.17–1.37	** <0.001**	0.82	0.69–0.98	**0.029**
SCOPA-PC	1.11	1.05–1.18	** <0.001**	–	–	–
Hallucinations^*^	6.70	3.31–13.55	** <0.001**	2.98	1.16–7.69	**0.023**
HADS, Anxiety	1.12	1.06–1.19	** <0.001**	0.9	0.81–1.01	0.09
HADS, Depression	1.21	1.14–1.29	** <0.001**	1.2	1.06–1.35	**0.002**

*OR, odds ratio; CI, confidence interval; SMS, Scales for Outcomes in Parkinson's disease-Motor Scale; ADL, activities of daily living; SCOPA-PC, Scales for Outcomes in Parkinson's disease-Psychiatric complications; HADS, Hospital Anxiety and Depression Scale*.

* *Hallucinations: item from SCOPA-PC. Bold indicates the significant values*.

Finally, in the multinomial model (Table 6), there was a positive association between the presence of PD-MCI and HY stage (OR, 2.45; 95% CI, 1.22–4.90). On the other hand, age (OR, 1.24; 95% CI, 1.07–1.44), HY stage (OR, 2.64; 95% CI, 1.17–5.98), and the depression HADS subscale (OR, 1.19; 95% CI, 1.02–1.37) were positively associated with the PDD group, whereas years of education (OR, 0.87; 95% CI, 0.78–0.97) was negatively associated. This model explained 61.6% of the variance.

**Table 6:** Multinomial logistic regression model (NC vs. PD-MCI vs. PDD).

	**PD-MCI**				**PDD**			
	**Crude OR**	***P***	**Adjusted OR (95% CI)**	***P***	**Crude OR**	***p***	**Adjusted OR (95% CI)**	***p***
Sex (women)	1.7	0.108	1.77 (0.83–3.80)	0.142	0.94	0.84	1.19 (0.49–2.88)	0.71
Age	1.01	0.682	0.99 (0.95–1.03)	0.705	1.15	** <0.001**	1.09 (1.04–1.16)	**0.001**
Years of education	0.9	**0.007**	0.93 (0.85–1.01)	0.097	0.79	** <0.001**	0.87 (0.78–0.97)	**0.014**
Disease duration	1.08	0.053	0.94 (0.82–1.07)	0.353	1.25	** <0.001**	1.13 (0.99–1.30)	0.065
Hoehn and Yahr	2.38	** <0.001**	2.45 (1.22–4.90)	**0.011**	9.65	** <0.001**	2.64 (1.17–5.98)	**0.02**
Barthel Index	0.92	** <0.001**	0.94 (0.91–0.98)	**0.008**	0.88	** <0.001**	0.93 (0.89–0.97)	**0.001**
SMS-Motor examination	1.1	**0.001**	1.07 (0.96–1.18)	0.191	1.26	** <0.001**	1.10 (0.99–1.22)	0.058
SMS-ADL	1.14	**0.007**	0.83 (0.69–1.00)	0.058	1.53	** <0.001**	0.88 (0.71–1.09)	0.244
SMS-Motor complic	1.15	**0.015**	0.99 (0.80–1.22)	0.938	1.38	** <0.001**	0.82 (0.65–1.03)	0.102
SCOPA-PC	0.94	0.192	–	–	1.08	**0.012**	–	–
Hallucinations^*^	1.58	0.477	0.87 (0.19–3.85)	0.858	8.76	** <0.001**	2.65 (0.63–11.08)	0.183
HADS, Anxiety	1.12	**0.003**	1.08 (0.95–1.23)	0.222	1.2	** <0.001**	0.96 (0.83–1.12)	0.664
HADS, Depression	1.1	**0.016**	1.00 (0.88–1.13)	0.970	1.29	** <0.001**	1.19 (1.02–1.37)	**0.021**

*OR, odds ratio; CI, confidence interval; SMS, Scales for Outcomes in Parkinson's disease-Motor Scale; ADL, activities of daily living; Motor complic, motor complications; SCOPA-PC, Scales for Outcomes in Parkinson's disease-Psychiatric Complications; HADS, Hospital Anxiety and Depression Scale*.

* *Hallucinations: item from SCOPA-PC. Bold indicates the significant values*.

## Discussion

This study applied the level I diagnostic criteria proposed by the MDS to analyze the differences between groups of PD patients according to their cognitive status. We found significant differences in sociodemographic, disease-related, and clinical variables depending on the severity of cognitive impairment, suggesting the usefulness of these criteria to classify PD-MCI and PDD patients according to their cognitive status. Recent studies reveal that levels I and II MCIs in PD classification have similar discriminative ability to predict the hazard of PDD (56). Nevertheless, previous studies suggest that level I criteria could be too broad and have a poor sensibility to classify PD patients; therefore, results should be interpreted carefully (57–59).

Participants with cognitive impairment (PD-MCI or PDD) were older and less educated than cognitively intact patients. In addition, they presented longer duration of PD, worse clinical and functional situation, higher levels of anxiety, depression, and greater presence of hallucinations than patients cognitively intact. Participants with cognitive impairment also had a worse quality of life and a more severe psychosocial impact of their disease, even in the early stages of cognitive decline (PD-MCI). Our findings are in line with other cross-sectional (24, 60, 61) and follow-up (24, 62) studies and highlight the importance of assessing these risk factors in the clinical setting. Moreover, non-motor symptoms have also been reported to affect the quality of life of PD patients to a greater extent than motor features, and this negative impact would appear from the very beginning of the disease (63).

Parkinson disease MCI and PDD patients were found to be more globally impaired than NC PD patients, with higher deterioration of both motor and non-motor symptoms, especially axial motor symptoms, depression, hallucinations, and cognitive performance measured by neuropsychological tests (64–66). All these features, considered even more disabling than the classic motor symptoms of PD, lead to a significant reduction in the quality of life of cognitive impaired patients.

A significant, although inverse, relationship between the degree of functionality (measured by the Barthel Index) and the presence of cognitive impairment was also found. This association, meaning that the less level of disability (corresponding to higher scores in Barthel index) is negatively associated with PD-MCI and PDD, compared to NC, is the only that consistently appeared in the binary and multinomial regression models. Multiple studies have already pointed out that dementia in PD results in a functional decline, and it has even been seen that the instrumental activities of daily life are also affected from the stage of MCI-PD (67). This relationship could be considered valid in both directions, because not only does cognitive impairment lead to greater disability, but also greater disability and physical frailty can eventually lead to “cognitive frailty,” meaning more decline in cognitive functions. As a matter of fact, these two conditions could share similar pathophysiological mechanisms, such as chronic inflammation, impaired hypothalamic–pituitary axis stress response, imbalanced energy metabolism, mitochondrial dysfunction, oxidative stress, and neuroendocrine dysfunction (68). A previous study has highlighted the important role of preserving cognitive functions to prevent disability and functional impairment (69).

The global severity of PD, measured by HY stage, and motor symptoms in PD were strongly associated with the presence of any type of cognitive impairment. Previous studies and reviews have shown that motor impairment is related to the overall presence of cognitive impairment (70, 71) and specifically to PD-MCI (72) and PDD (73, 74). In line with these previous studies, we found that the presence of cognitive impairment was associated with a more severe HY stage of PD. Conversely, the inverse association between motor complications and the presence of dementia that was found in one of our logistic regression models is not consistent with the reviewing literature that report motor complications as a risk factor for the development of PDD (24, 75). However, there is limited evidence on this topic, and the short length of the subscale that we have used (only four items, two for dyskinesias, and two for motor fluctuations) in combination with the lack of information on patient's treatment could explain a negative association between dementia and motor complications. Moreover, one study found that dyskinesias, but not motor fluctuations, were significantly related to dementia (24).

Our findings suggest that PD-MCI and PDD patients showed more neuropsychiatric symptoms than the NC patients. In the vast majority of studies, with different rating scales, neuropsychiatric symptoms seem to be related to cognitive impairment (74). Moreover, these disruptions could independently affect to disease progression by means of damaging frontal–subcortical pathways (76). Our study has found that behavior disorders are highly common in PD, and as suggested in previous studies, there may be a certain overlap between this symptomatology and cognitive symptoms (77).

We found a high prevalence of anxiety, depression, and hallucinations in PDD patients. Studies have shown that these features occur in ~30% of PD patients, often in the early stage of the disease (3). In binary logistic regression models, a statistical association between the last two and the presence of PDD was also found, in agreement with previous studies (12, 66). Visual hallucinations have been proposed as predictors of future development of dementia in patients with PD (12, 22). With the presence of cognitive dysfunction emerges the possibility of a higher frequency of hallucinations and depressive symptomatology. Studies indicate that both dementia and visual hallucinations share the same limbic system networks (24). Moreover, depression in PD has been associated to decreased white matter in the fornix, as well as cognitive impairment and apathy (78), and differences in brain circuitry appear since mild stages of depression (79).

Higher age and low educational level are well-known risk factors in the general population for developing cognitive impairment and dementia (24, 71), and the same is true for PDD (30, 36, 41, 49), as shown in our study. According to some authors, advanced age in PD is associated with a much higher risk of dementia than the effect of age alone (24), suggesting some kind of interaction effect between age and severity of PD in the risk of dementia (60). On the other hand, the relationship between age, educational level, and PD-MCI is not completely clear (72) but higher educational level seems to be a protective factor in recent studies, in line with our trends (80, 81).

Our results did not find association between gender and cognitive impairment, in agreement with previous studies (66, 72). As women are older than men when they start with the symptoms, they have a shorter disease duration, compared with men of the same age. Therefore, men with PD would have a more advanced disease stage than women of the same age, increasing their risk of PDD (71). However, there are also studies that have found an association between male sex and PDD/MCI-PD after controlling for age and disease duration (21, 65, 82), so the significance of this risk factor remains unclear.

Regarding the disease-related risk factors, a positive relationship between PD duration and PDD was found. However, age is also related to the duration of the disease, and for that reason, it is difficult to unravel the effects of each one on the risk of dementia. Furthermore, a very recent cross-sectional study assessing dementia in long-term PD patients did not find any significant differences between age, age at onset, or disease duration between PD patients with and without dementia (83).

Several limitations of this study should be acknowledged. Although we carried out the assessment during “on” state, cognitive fluctuations are always possible in PD. Because we performed a single evaluation over time, and PRO scales (HADS, SCOPA-PS and PDQ-8) in cognitive impairment patients were completed by caregivers (proxy evaluations), the results have to be taken with caution and should not be interpreted as causal. In addition, we do not know the premorbid performance or cognitive reserve of the participating patients, and the results of cognitive scales could have been affected by these factors.

Our study, designed for a broader goal, did not allow applying a completely exhaustive neuropsychological evaluation. Therefore, only the “possible” PD-MCI and PDD status could be achieved. However, all the applied tests have been widely validated in the literature, and in particular, the screening test used, SCOPA-Cog, was proposed by the MDS as a scale of global cognitive abilities validated for use in PD, whose clinimetric characteristics are satisfactory (84). This fact increases the reliability of the finding results.

At last, we would like to remark that we did not carry out a cluster analysis in this study, as there is no widely accepted consensus of how best to group patients (85). Yet, the criteria to identify subtypes and predict individual prognosis remain unclear and therefore lack clinical applicability and reproducibility (30). Moreover, differences in inclusion criteria from datasets, variable selection, and methodology between studies using cluster analysis have made it difficult to compare the subtypes, and the features describing them can be confusing and overlapping (86). Most of studies are cross-sectional, with a short follow-up, so they are often considered of limited utility (87). Finally, the division of PD into major subtypes has been criticized because it could be a simplification of the heterogeneous reality in the disease (36).

In this study, cognitive decline was associated with a worse disease stage at a global, psychiatric, and psychosocial level and therefore with an impairment of quality of life. We also observed that the patients with greater physical disability had worse cognitive functioning, so it seems important to identify the progression of disease to prevent cognitive impairment.

Some patient characteristics, such as age and lower educational level, were independently associated with dementia, as reported in previous studies. These data could help to understand the deeply impact that cognitive decline has on PD prognosis and highlight the importance of design and deliver integrated care for PD patients and their families. The greater knowledge on non-motor features would undoubtedly lead to a more accurate PD diagnosis and better treatments. As a result, the quality of life of these patients and the living conditions of their caregivers and family members would also improve. We strongly recommend assessing cognitive status at the time of PD diagnosis; exploring premorbid cognitive status, appearance, and type of deficits; and monitoring changes in disease severity over time. It is also relevant to pay special attention to neuropsychiatric symptoms, mainly depression and presence of hallucinations, as they seem to be strongly associated with cognitive impairment. Nevertheless, further research is required to understand the underlying pathophysiological mechanisms that link cognitive impairment with the remaining non-motor symptoms in PD.

## Data Availability Statement

The raw data supporting the conclusions of this article will be made available by the authors, without undue reservation.

## Ethics Statement

Ethical review and approval was not required for the study on human participants in accordance with the local legislation and institutional requirements, as all datasets were fully anonymized prior to transfer and processing. Our paper is a retrospective (secondary) study, not requiring review and approval.

## Author Contributions

PM-M initiated and designed the study, and collected the data within the Cog-PD study. AS-G, CR-B, and MF conducted the data analysis and wrote the main manuscript text. All authors reviewed the manuscript.

## Conflict of Interest

The authors declare that the research was conducted in the absence of any commercial or financial relationships that could be construed as a potential conflict of interest.
